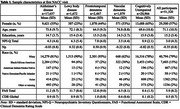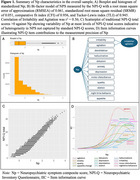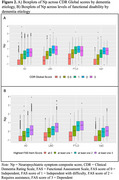# Bi‐factor Model of Neuropsychiatric Symptom Measurement in National Alzheimer's Coordinating Center

**DOI:** 10.1002/alz70857_106660

**Published:** 2025-12-25

**Authors:** Emma Rhodes, Rich Jones, Sheina Emrani, Paul K Crane, Seo‐Eun Choi

**Affiliations:** ^1^ Penn Frontotemporal Degeneration Center, Department of Neurology, Perelman School of Medicine, University of Pennsylvania, Philadelphia, PA, USA; ^2^ Brown University, Providence, RI, USA; ^3^ Department of General Internal Medicine, University of Washington School of Medicine, Seattle, WA, USA

## Abstract

**Background:**

Neuropsychiatric symptoms (NPS) are nearly universal in dementia, but measurement limitations have hindered progress in understanding their pathophysiology and mechanisms. NPS are commonly assessed using the Neuropsychiatric Inventory–Questionnaire (NPI‐Q), a 12‐item informant‐report survey designed to capture the presence and severity of NPS in ADRD. Despite its popularity, utility of the NPI‐Q is limited by problems with measurement precision and unclear dimensionality. The present study investigated potential to improve measurement of NPS with the NPI‐Q using bi‐factor model analysis.

**Method:**

Data from 51,520 participants with all‐cause dementia or unimpaired cognition were obtained from NACC. For each participant we selected the occasion at which they had their highest total NPI‐Q total score. Confirmatory factor analysis (CFA) assessed dimensionality of NPS in the overall sample and across dementia etiologies with >500 participants including Alzheimer's disease (AD), Lewy body dementia (LBD), frontotemporal dementia (FTLD), and vascular dementia (VaD). Composite factor scores (Np) were derived for all timepoints and compared against NPI‐Q total scores. Item information curves assessed the independent contribution of NPI‐Q items to the precision of Np in the overall sample. Associations of Np with baseline disease severity measured by the Clinical Dementia Rating Scale (CDR) and functional disability characterized by the highest level of functional dependence reported for each participant on the Functional Assessment Scale (FAS) were explored across dementia groups.

**Result:**

CFA of NPI‐Q scores resulted in a bi‐factor model of NPS with a secondary factor of Irritability/Agitation (Figure 1B). Correlation of Np with NPI‐Q total was 0.94 with variability of Np at most levels of NPI‐Q total scores (Figure 1C). Baseline Np was highest in FTLD and LBD relative to other dementia groups, and Np in all dementia groups was greater than unimpaired controls (Table 1). Higher Np was observed in moderate‐severe disease and associated with greater functional disability across all dementia groups (Figure 2).

**Conclusion:**

Bi‐factor model scoring of the NPI‐Q in NACC highlights variability in NPS in ADRD obscured by the NPI‐Q Total score. Np scores may provide important information from NPI‐Q data across a variety of dementia diagnoses.